# Tumor-Like Lesions in the Craniovertebral Junction: A Case Series, Systematic Review, and Meta-Analysis

**DOI:** 10.3390/cancers16162788

**Published:** 2024-08-07

**Authors:** Maria Mihaela Pop, Dragos Bouros, Artsiom Klimko, Laura Ancuta Pop, Paula Topal, Anil Topal, Ioan Stefan Florian

**Affiliations:** 1 Department of Neurosurgery, Iuliu Hatieganu University of Medicine and Pharmacy, 400012 Cluj-Napoca, Romania; stefanfloriannch@gmail.com; 2 Clinic of Neurosurgery, Cluj County Emergency Clinical Hospital, 400347 Cluj-Napoca, Romania; topalpaula.md@yahoo.com; 3 Laboratory of Molecular Neuro-Oncology, Department of Neurology, University Hospital Zurich, 8091 Zürich, Switzerland; artsiom.klimko@usz.ch; 4 Research Center for Functional Genomics, Biomedicine and Translational Medicine, Iuliu Hatieganu University of Medicine and Pharmacy, 400012 Cluj-Napoca, Romania; laura.ancuta.pop@gmail.com; 5 Faculty of Medicine, Inonu University, 44000 Malatya, Turkey; a.topal.med@hotmail.com

**Keywords:** craniovertebral junction (CVJ), tumor-like lesions, neurenteric cyst, epidermoid cysts, dermoid cysts, systematic review, meta-analysis

## Abstract

**Simple Summary:**

Tumor-like lesions at the craniovertebral junction, including epidermoids, dermoids, and neurenteric cysts present significant treatment challenges. Our review of several cases, combined with a meta-analysis of numerous patients, aimed to identify recurrence predictors to improve treatment and follow-up accuracy. Neurenteric cysts were the most common, generally presenting with gradual symptom onset over several months and commonly located in the anterior part of the CVJ. Recurrence in neurenteric cyst cases was linked to factors such as older age, subtotal resection, complications, rim enhancement, and poor outcomes. Recognizing these predictors is essential for enhancing treatment strategies and patient management. By understanding the key factors influencing recurrence, healthcare professionals can better tailor their approaches to ensure optimal outcomes for patients with CVJ lesions.

**Abstract:**

**Background**: Tumor-like lesions at the craniovertebral junction mimic tumors in clinical presentation and imaging. Our study focuses on three common developmental pathologies—epidermoids, dermoids and neurenteric cysts. **Methods**: We conducted a retrospective analysis of a case series and a meta-analysis of 170 patients from 119 reports. **Results**: Neurenteric cysts predominated (81.2%). Anterior cysts were linked to neurenteric cysts, while posterior ones correlated with dermoid/epidermoid cysts (*p* < 0.001). Complications occurred in 27.2% of cases, with cranial nerve paresis being the most common. Most patients had excellent outcomes (75.2%) with low recurrence rates (12%). Dermoid cysts were more associated with anomalies (*p* < 0.001). Among 138 neurenteric cyst cases, 15 experienced recurrence, with predictors including ages 51–60 and over 70, subtotal resection, complications, and poor outcomes (*p* < 0.001). Cysts with total resection were significantly less likely to adhere to surrounding brain tissue (*p* < 0.001). CSF diversion was correlated with older age (*p* = 0.010) and various complications (*p* < 0.001). Age affected outcomes, and the hydrocephalus was linked to poor outcomes (*p* = 0.002). **Conclusions:** This meta-analysis underscores the importance of total resection in minimizing recurrence rates and emphasizes meticulous preoperative planning and imaging. Our results indicate that rim enhancement (*p* = 0.047) and poor outcome (*p* = 0.007) are significant factors associated with recurrence. Additionally, associated anomalies, as well as the patient’s age and overall health, significantly influence the surgical outcomes and the likelihood of recurrence.

## 1. Introduction

Tumor-like lesions of the craniovertebral junction (CVJ) are relatively rare and encompass a variety of pathologies, including neoplastic, congenital, and acquired lesions. The CVJ is an anatomically complex area comprising the clivus, foramen magnum, and atlantoaxial complex, presenting significant challenges for surgical management due to the critical neurovascular structures and limited surgical corridors available for safe resection.

Tumor-like lesions refer to non-neoplastic pathological entities that mimic tumors in their clinical presentation and imaging characteristics. The World Health Organization (WHO) classifies dermoids, epidermoids, and neurenteric cysts as malformations and tumor-like lesions that can occur anywhere in the brain, including the CVJ. These lesions can appear similar to true tumors on imaging but are of different pathological origins and behaviors. Neurenteric cysts, for instance, are congenital lesions arising from the remnants of the notochord and endoderm, typically located ventrally to the brainstem and spinal cord and often containing mucinous material. On the other hand, epidermoid cysts are slow-growing congenital tumors originating from ectodermal inclusions and are filled with desquamated keratin; these cysts can become symptomatic due to mass effect or inflammation. Dermoid cysts, similar to epidermoid cysts, contain ectodermal elements such as hair follicles and sebaceous glands and can cause mass effect or rupture, leading to chemical meningitis. Differential diagnoses for CVJ lesions include tumoral conditions such as meningiomas, schwannomas, and chordomas, as well as non-tumorous conditions like arachnoid cysts and inflammatory granulomas. Accurate preoperative imaging and histopathological analysis are essential for distinguishing these entities and ensuring appropriate management [1,2,3,4].

The surgical management of these lesions often involves a posterior fossa craniotomy or craniectomy with a midline suboccipital approach. The transoral route has traditionally been the standard approach for ventral lesions, but it carries significant morbidity [5]. Advances in endoscopic techniques have led to the development of the endoscopic endonasal approach (EEA) for ventral CVJ lesions. This approach avoids the complications associated with the transoral route and provides a direct, less traumatic path to the target area.

Surgery aims to achieve the maximal safe resection of the lesion while preserving neurological function. Intraoperative neuromonitoring, including somatosensory evoked potentials (SSEPs), motor evoked potentials (MEPs), and cranial nerve monitoring, is often employed to minimize the risk of iatrogenic injury. Given the complexity of the CVJ and the potential for significant morbidity, a multidisciplinary team approach is essential for managing these lesions. This team should involve neurosurgeons, otolaryngologists, neuroradiologists, and neuro anesthesiologists. Preoperative imaging, including MRI and CT, is critical for surgical planning and understanding the relationship of the tumor-like lesions to surrounding structures [6,7,8,9,10,11,12,13,14].

To our knowledge, this is the first systematic review and meta-analysis providing a retrospective review of clinical information, imaging features, surgical details, and follow-up outcomes of such cases reported in the literature between 1984 and 2024. Additionally, we describe seven cases that were presented consecutively to our institution from 2012 until December 2023.

## 2. Materials and Methods

### 2.1. Personal Case Series

A retrospective analysis was conducted over a 12-year period for patients admitted to the Department of Neurosurgery at Cluj-Napoca County Emergency Hospital, spanning from 2012 to 2023. During this time, 48 patients with developmental CVJ anomalies were treated at our institution, of whom seven harbored congenital tumor-like lesions. All adult patients and the guardians of pediatric patients provided informed consent for both the intervention and participation in this study. All operations were performed by the same neurosurgeon, ensuring consistency in surgical technique.

Ethical approval: This retrospective study involving human participants was in accordance with the ethical standards of the institutional and national research committee and with the 1964 Helsinki Declaration and its later amendments or comparable ethical standards. The Iuliu Hatieganu University of Medicine and Pharmacy, Cluj-Napoca, Research Ethics Committee has confirmed that no ethical approval is needed.

### 2.2. Literature Search Strategy

This study used the PRISMA (Preferred Reporting Items for Systematic Reviews and Meta-Analyses) guidelines and recommendations. This systematic review has been registered in PROSPERO with the registration number 572,501 [15]. A systematic literature search of the English literature from 1984 to 2024 was performed in the PubMed, SCOPUS, Web of Science, and EMBASE databases on 26 February 2024. The search focused on studies on neurenteric, epidermoid, and dermoid cysts in the CVJ. The search was not limited by study design and included English keywords; thus, at least the abstract had to be in English. The search protocol was designed using various medical subject headings (MeSH) and keywords related to neurenteric, epidermoid, or dermoid cysts and the CVJ or its synonyms. All records retrieved from these databases were imported into Excel, where duplicate records were detected and excluded. The following search terms were used: (“neurenteric cyst” OR “neurenteric tumor” OR “endodermal cyst” OR “endodermal tumor” OR “enterogenous cyst” OR “enterogenous tumor” OR “epithelial cyst” OR “epithelial tumor” OR “teratomatous cyst” OR “teratomatous tumor” OR “enterogenic cyst” OR “enterogenic tumor” OR “foregut cyst” OR “foregut tumor” OR “respiratory cyst” OR “respiratory tumor” OR “bronchogenic cyst” OR “bronchogenic tumor” OR “intestinoma cyst” OR “intestinoma tumor” OR “gastrocytoma cyst” OR “gastrocytoma tumor” OR “dermoid cyst” OR “dermoid tumor” OR “dermoids” OR “epidermoid cyst” OR “epidermoid tumor” OR “epidermoids”) OR ((“neurenteric” OR “endodermal” OR “enterogenous” OR “teratomatous” OR “enterogenic” OR “foregut” OR “bronchogenic” OR “intestinoma” OR “gastrocytoma” OR “dermoid” OR “epidermoid”) AND (“cyst” OR “tumor”)) AND (“cervicomedullary junction” OR “cervico-medullary junction” OR “CMJ” OR “craniovertebral junction” OR “cranio-vertebral junction” OR “CVJ” OR “craniospinal junction” OR “cranio-spinal junction” OR “CSJ” OR “craniocervical junction” OR “cranio-cervical junction” OR “CCJ” OR “foramen magnum” OR “C1” OR “atlas” OR “first cervical vertebra” OR “C2” OR “axis” OR “second cervical vertebra” OR “C1-C2” OR “C1 and C2” OR “upper cervical spine”), from 1984 to 2024. Additionally, cited references from relevant review articles were also reviewed.

### 2.3. Eligibility Criteria

Two authors independently screened the articles, and two additional authors resolved any disagreements. Articles meeting the following criteria were included in this study: (1) cases located in the CVJ; (2) confirmed cases of epidermoid, dermoid, or neurenteric cysts managed by surgical resection; (3) human studies; (4) cases published from 1984 onwards.

Studies were excluded if they met any of the following criteria: (1) patients did not undergo surgery; (2) cadaveric studies; (3) animal studies or in vitro experiments; (4) articles that were inaccessible; (5) articles in languages other than English that could not be elucidated from the available English segment; (6) insufficient data reported. Letters, commentaries, editorials, and expert opinions were also excluded.

### 2.4. Screening and Data Extraction

The screening was based on publication titles and abstracts, with full texts of remaining publications obtained for eligibility assessment and data extraction. Parameters collected, where available, included author, year, age, sex, histopathology, symptoms/signs, duration of symptoms, tumor location, cistern location, CT, and MRI characteristics, craniotomy, adhesion to surrounding brain, extent of resection, complications, associated anomalies, outcome, recurrence, and follow-up period.

### 2.5. Data Analysis

Data were analyzed using IBM SPSS Statistics 25 (IBM Corp., Armonk, NY, USA) and illustrated using Microsoft Office Excel/Word 2021 (Microsoft Corp., Redmond, WA, USA). Quantitative variables were tested for normal distribution using the Shapiro–Wilk Test and were written as averages with standard deviations or medians with interquartile ranges. Qualitative variables were written as counts or percentages and differences between groups were tested using Fisher’s exact test. Z-tests with Bonferroni correction were used to detail the results obtained in the contingency tables.

Quantitative independent variables with non-parametric distribution were tested between groups using Mann–Whitney U/Kruskal–Wallis H tests (according to analyzed groups). Quantitative independent variables with normal distribution were tested between groups using Student *t*-tests/Welch-T-Test/One-Way ANOVA tests/Welch One-Way ANOVA tests (according to homogeneity of variances and analyzed groups). Post hoc Dunn–Bonferroni tests were used to further detail the results obtained from testing the quantitative independent variables.

Univariable and multivariable logistic regression models were used to calculate the effect of different risk factors over recurrence. Models were tested for significance and goodness-of-fit and the prediction effect was estimated as odds ratios with 95% confidence intervals. The threshold considered for the significance level for all tests was considered to be α = 0.05.

## 3. Results

### 3.1. Personal Case Series (Table 1)

Case 1: A 30-year-old female presented with insidious symptoms of headache, neck pain, and quadriparesis over two years. MRI revealed an intradural extramedullary tumor at the posterior CVJ, extending from the cerebellomedullary cistern down to the C1 vertebra, characterized by T1 hyperintensity and T2 hypointensity, with no restriction on diffusion-weighted imaging (DWI) and no enhancement. The patient underwent a craniotomy with C1 laminectomy, achieving the successful surgical excision of the tumor via a posterior midline approach in the prone position, without complications despite the tumor’s adherence to surrounding brain tissue. The histopathological analysis identified a neurenteric cyst.

**Figure 1:**
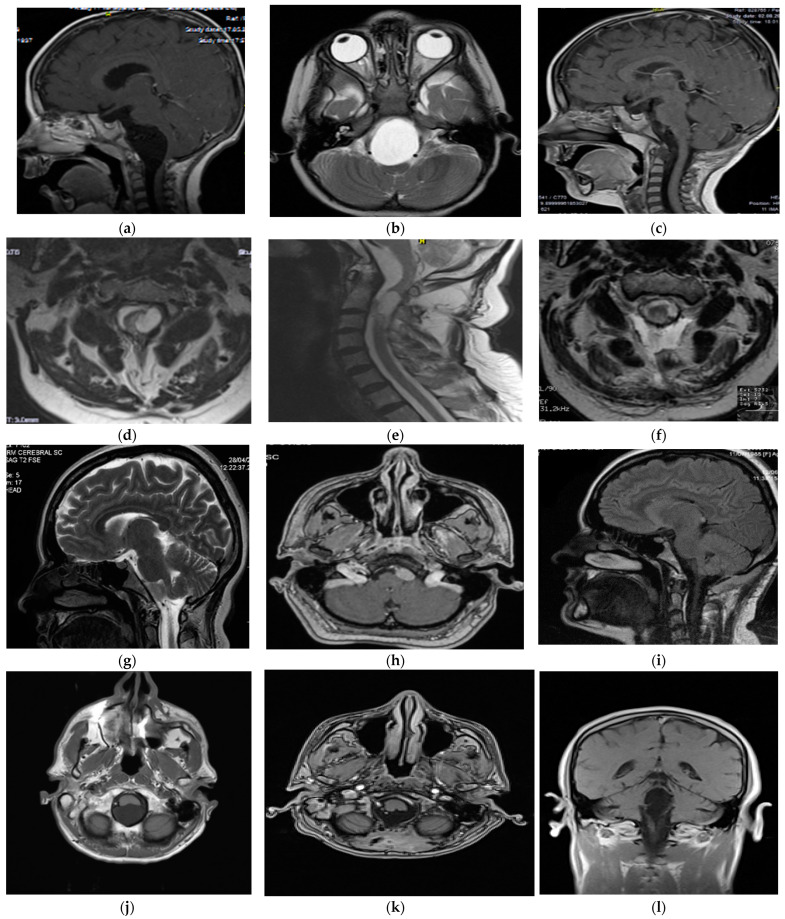
Preoperative and postoperative MRI characteristics of personal case series. Patient #2 (CVJ neurenteric cyst)—preoperative MRI (image (**a**)—saggital cut, T1-weighted; image (**b**)—axial cut, T2-weighted) showing a hypointense mass in the pontomedullary cistern extending to C1; 3-month follow-up MRI (image (**c**)) demonstrating the extent of the gross total resection. Patient #3 (CVJ dermoid cyst)—preoperative MRI (image (**d**)—axial cut, T2-weighted; image (**e**)—saggital cut, T2-weighted) showing a hyperintense mass in the premedullary cistern extending to C2; 3-month follow-up MRI (image (**f**)—axial cut, T2-weighted) demonstrating extent of subtotal resection. Patient #4 (CVJ neuroenteric cyst)—preoperative MRI (image (**g**)—saggital cut, T2-weighted; image (**h**)—axial cut, T1-contrast) showing a hypointense mass without enhancement in the premedullary region; 3-month follow-up MRI (image (**i**)—saggital cut, T1-weighted) demonstrating the extent of gross total resection. Patient #6 (CVJ epidermoid cyst) — preoperative MRI (image (**j**)—axial cut, T2-weighted) revealed a hyperintensity mass in the cerebellomedullary cistern extending to C1; 3-month follow-up MRI (image (**k**)—axial cut, T1 contrast, image (**l**)—coronal cut, T1-weighted) demonstrate gross-total resection.

Case 2: A 3-year-old boy experienced insidious symptoms of aseptic meningitis over one year, leading to the discovery of an anterior CVJ tumor located intradural, extending from the pontomedullary cistern down to the C1 vertebra. MRI characteristics included T1 hypointensity and T2 hyperintensity, with no restriction on DWI and no enhancement (Figure 1). Histopathological analysis confirmed a neurenteric cyst. Surgical excision was performed via a lateral suboccipital craniotomy with C1 hemilaminectomy in the sitting position, utilizing a far-lateral approach due to the cyst’s location, resulting in total excision with no complications reported. Long-term follow-up over 12 years showed no recurrence, with an excellent outcome.

Case 3: A 73-year-old male presented with a 15-month history of slowly developing symptoms of headache, diplopia, and gait disturbances, leading to the discovery of an anterior and lateral CVJ dermoid cyst extending from the premedullary cistern down to the C2 vertebra. MRI showed T1 hypointensity and T2 hyperintensity, with no restriction on DWI and no enhancement (Figure 1). Surgical excision was performed via a lateral suboccipital craniectomy with C1 laminectomy, utilizing a far-lateral approach in the sitting position. The patient had an uneventful postoperative course, with subtotal resection of the cyst achieved. Long-term follow-up spanning 8 years showed an excellent outcome with no evidence of cyst recurrence.

Case 4: A 34-year-old female presented with an insidious headache as the primary symptom. MRI revealed an anterior and lateral CVJ neurenteric cyst located intradural, with a cistern location in the premedullary region, characterized by T1 hyperintensity and T2 hypointensity, with no enhancement (Figure 1). Surgical excision in the sitting position was performed via a lateral suboccipital craniectomy with C1 laminectomy, utilizing a far-lateral approach. Total cyst resection was achieved, and postoperative imaging confirmed complete excision. One-year follow-up showed an excellent outcome with no recurrence.

Case 5: A 73-year-old male presented with an 8-month history of insidious symptoms, including headache, gait disturbance, and ataxia, leading to the discovery of a posterior and lateral CVJ epidermoid cyst located intradural, extending from the cerebellomedullary cistern down to the C1 vertebra. MRI showed no enhancement in T1 hypointensity, T2 hyperintensity, and hyperintensity on DWI. Surgical excision in the sitting position via a retromastoid craniotomy with a retrosigmoid approach resulted in the subtotal resection of the cyst, with no complications reported. Postoperative imaging showed no evidence of cyst recurrence, and the patient had a 3-year follow-up.

Case 6: A 53-year-old male presented with a two-year history of insidious symptoms, including headache, gait disturbance, ataxia, and diplopia. Imaging revealed a posterior CVJ epidermoid cyst extending from the cerebellomedullary cistern down to the C1 vertebra. MRI showed T1 hypointensity, T2 hyperintensity, and hyperintensity on DWI, with no enhancement (Figure 1). Surgical excision in the sitting position via a midline suboccipital craniotomy resulted in the total resection of the cyst, with no complications reported. Postoperative imaging confirmed complete excision, and the patient underwent a 6-year follow-up with an excellent outcome.

Case 7: A 22-year-old male presented with sudden symptomatic hydrocephalus and dizziness over 5 days. Imaging revealed a posterior CVJ epidermoid cyst located intradural in the cerebellomedullary cistern. MRI demonstrated no enhancement of T1 hypointensity, T2 hyperintensity, and hyperintensity on DWI. Surgical excision in the sitting position using a posterior midline approach resulted in total cyst resection. Postoperative imaging confirmed complete excision, although the patient experienced persistent hydrocephalus requiring a ventriculoperitoneal (VP) shunt. Despite this, the patient had a good outcome at a 5-year follow-up, with no late complications observed.

**Table 1:** Personal case series patients’ characteristics.

Case	Age/Sex	Symptoms	Duration	Location	MRI Characteristics	Surgical Approach	Histopathology	Outcome	Follow-up
1	30/F	Headache, neck pain, quadriparesis	2 years	Posterior CVJ, cerebellomedullary cistern to C1	T1 hyperintensity, T2 hypointensity, no DWI restriction, no enhancement	Craniotomy with C1 laminectomy, posterior midline approach	Neurenteric cyst	No complications, complete excision	5 years, no recurrence
2	3/M	Aseptic meningitis	1 year	Anterior CVJ, a pontomedullary cistern to C1	T1 hypointensity, T2 hyperintensity, no DWI restriction, no enhancement	Lateral suboccipital craniotomy with C1 hemilaminectomy, far-lateral approach	Neurenteric cyst	No complications, total excision	12 years, no recurrence
3	73/M	Headache, diplopia, gait disturbances	15 months	Anterior and lateral CVJ, premedullary cistern to C2	T1 hypointensity, T2 hyperintensity, no DWI restriction, no enhancement	Lateral suboccipital craniectomy with C1 laminectomy, far-lateral approach	Dermoid cyst	Uneventful, subtotal resection	8 years, no recurrence
4	34/F	Headache	-	Anterior and lateral CVJ, premedullary region	T1 hyperintensity, T2 hypointensity, no enhancement	Lateral suboccipital craniectomy with C1 laminectomy, far-lateral approach	Neurenteric cyst	No complications, total resection	1 year, no recurrence
5	73/M	Headache, gait disturbance, ataxia	8 months	Posterior and lateral CVJ, cerebellomedullary cistern to C1	T1 hypointensity, T2 hyperintensity, hyperintensity on DWI, no enhancement	Retromastoid craniotomy, retrosigmoid approach	Epidermoid cyst	No complications, subtotal resection	3 years, no recurrence
6	53/M	Headache, gait disturbance, ataxia, diplopia	2 years	Posterior CVJ, cerebellomedullary cistern to C1	T1 hypointensity, T2 hyperintensity, hyperintensity on DWI, no enhancement	Midline suboccipital craniotomy	Epidermoid cyst	No complications, total resection	6 years, excellent outcome
7	22/M	Hydrocephalus, dizziness	5 days	Posterior CVJ, cerebellomedullary cistern	T1 hypointensity, T2 hyperintensity, hyperintensity on DWI, no enhancement	Posterior midline approach	Epidermoid cyst	Persistent hydrocephalus (VP shunt), good outcome	5 years, no late complications

### 3.2. Search Strategy

**Figure 2:**
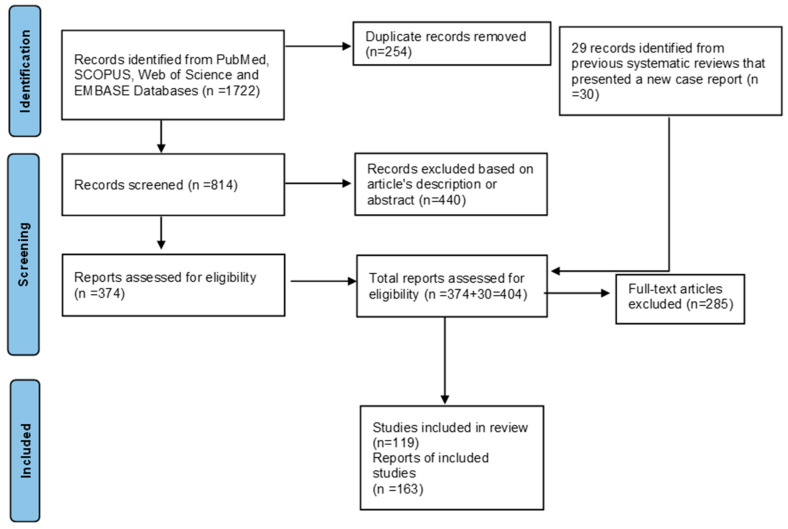
PRISMA 2020 flow diagram.

The initial search identified a total of 1722 references across the four databases. After removing duplicates, 814 unique studies underwent the initial title and abstract screening process. Ultimately, 374 articles were considered potentially eligible and retrieved for complete analysis. Additionally, we reviewed all 29 previous systematic reviews that included new case reports, which yielded 30 publications for full-text analysis. In total, 404 full-text articles were reviewed and assessed. Finally, 119 studies met the inclusion criteria and were incorporated into our analysis. A PRISMA diagram illustrating the data selection and processing is shown in Figure 2.

### 3.3. Patient Baseline Characteristics for Tumor-Like Lesions

The current meta-analysis included 170 patients (163 from the literature—95.88% and 7 from our case series—4.12%). Neurenteric cysts constituted 81.2% of cases—138 cases; dermoid cysts accounted for 11.2%—19 cases; and epidermoid cysts represented 7.6% of the cohort—13 cases. The gender distribution was nearly equal, with 48.8% male patients—83 patients. The median age was 27 years, with the most frequent age categories being 5–18 years (24.1%—41 patients) and 19–30 years (22.9%—39 patients), as seen in Table 2.

The most frequent symptoms were headaches (56.9%—95 patients), neck pain (35.1%—59 patients), head and neck movement limitation (17.3%—29 patients), sensory abnormalities (17.3%—29 patients), meningitis (13.1%—22 patients), cardio-respiratory events (3.6%—6 patients); and bladder and bowel involvement (2.4%—4 patients); head and neck movement limitation (*p* = 0.032); and meningitis (*p* = 0.049) were more frequently associated with dermoid cysts than with neurenteric cysts. Paresis (29.2%—49 patients), particularly quadriparesis (15.5%—26 patients), cerebellar signs (19.8%—33 patients), lower cranial nerve paresis (17.4%—29 patients), nausea/vomiting (15.5%—26 patients), and symptomatic hydrocephalus (7.1%—12 patients) were also observed as frequent symptoms, cerebellar signs (*p* < 0.001) and symptomatic hydrocephalus (*p* = 0.030) being more frequent in patients with epidermoid cysts than those with neurenteric cysts. Most patients reported a gradual onset of symptoms (87.5%—126 patients), with a median duration of 5 months (IQR = 1.25–12); 51.2% of patients—64 patients experienced symptoms lasting between 1 month and 1 year.

The tumor location at the CVJ was documented in 117 articles: anterior (35.7%—60 patients), anterolateral (36.3%—61 patients), or posterior CVJ (19.6%—33 patients). Tumors located anteriorly were significantly more associated with neurenteric cysts than dermoid cysts, while anterolateral tumors were more commonly associated with neurenteric cysts than epidermoid cysts. Posterior CVJ tumors were significantly more associated with dermoid or epidermoid cysts than neurenteric cysts (*p* < 0.001). Regarding cistern anatomical distribution, most cysts were located in the premedullary cistern (15%—25 patients), extending down towards the spinal canal (25.7%—43 patients), or within the cerebellomedullary cistern (13.8%—23 patients), with some extending into the spinal canal (12.6%—21 patients). Cysts at the cerebellomedullary cistern were significantly more associated with dermoid or epidermoid cysts than neurenteric cysts (*p* < 0.001). At the same time, those extending towards the spinal canal were significantly more associated with epidermoid cysts (*p* < 0.001).

Among the 48 patients for whom CT data were available, 45.8%—22 patients had a hypodense aspect, with a median cyst volume of 14.86 mL (IQR = 6.26–40.16 mL). The MRI characteristics of 129 patients were described as homogenous in 71.3% of cases—92 cases, with T1 hypersignal (37.9%—53 cases) or hyposignal (38.6%—54 cases) and T2 hypersignal (69.5%—89 cases). Most cysts on DWI showed no restriction (83.3%—30 cases), and data from 108 patients indicated no contrast enhancement (68.5%—74 patients). According to the type of craniotomy and surgeon preference, 38.3% of cases—23 cases were operated on in the lateral position; 35%—21 cases in the prone position; 21.7%—13 cases in the sitting position; and 5%—3 cases in the supine position. For cysts located in the anterior or anterolateral portion of the CVJ, a far-lateral (25.2% of cases—34 cases) or a far-lateral transcondylar approach (17% of cases—23 cases) was used. The lateral suboccipital (retrosigmoid) craniotomy was performed in 17% of cases—23 cases, more commonly for epidermoid cysts than dermoid cysts (*p* = 0.002). The most frequent approach was the posterior midline approach for cysts located in the posterior CVJ or those extending into the cervical spinal canal (37.8%—51 cases), which was significantly more associated with dermoid cysts than neurenteric cysts (*p* = 0.002). In four cases (3%), the transoral route was used for cysts in the anterior or anterolateral CVJ. Despite adhesions to surrounding brain tissue in 70.7% of cases—87 cases—complete excision was feasible in 63.7%—100 cases—of cases. Near-total excision was performed in 10.8% of cases—17 cases—while in 25.5% of cases—40 cases—a significant portion of the cyst wall was left behind due to its close adherence to neurovascular structures.

The rate of complications was 27.2%—41 cases—with the most common permanent postoperative complication being cranial nerve paresis (9.27%—14 cases). The median follow-up period was 22 months (IQR = 6–40.5 months). 

To provide clarity on patient outcomes, we categorized them using validated scales: (1) Excellent outcomes: defined as scores of 90–100 on the Karnofsky performance scale (KPS) or 0–1 on the modified Rankin scale (mRS), indicating that the patient is able to carry out normal activities with no symptoms or minor symptoms; (2) Good outcomes: defined as scores of 80–89 on the KPS or 2 on the mRS, indicating slight disability but still able to look after own affairs without assistance; (3) Bad or poor outcomes: defined as scores below 80 on the KPS or 3–6 on the mRS, indicating varying degrees of disability, dependence, or mortality. Higher scores on the mRS represent more severe disability or death. These scales provide a standardized measure for assessing postoperative recovery and long-term functionality, allowing for consistent evaluation across studies. Most patients had excellent outcomes (75.2%—112 cases) with low recurrence rates (12%—15 cases). In recurrent cases, two instances of malignant transformation were found. Additionally, 17.6%—29 cases—of cysts had associated anomalies, which were significantly more associated with dermoid cysts than neurenteric or epidermoid cysts (*p* < 0.001).

### 3.4. The Meta-Analysis of Neurenteric Cyst: Prognostic Factors for Recurrence

Among the 138 cases of neurenteric cysts, 13 patients (12.4%) experienced local recurrence, which correlated with several clinical parameters. Statistical analysis revealed that patients aged 51–60 and over 70 were significantly more associated with recurrence (*p* = 0.005). The presence of symptomatic hydrocephalus (*p* = 0.025) and the need for CSF diversion (*p* < 0.001) were also significantly correlated with recurrence. Imaging characteristics such as T1 hyposignal (*p* = 0.007) and rim enhancement (*p* = 0.002) were significant prognostic factors. Other prognostic factors included subtotal resection (*p* < 0.001), postoperative complications (*p* < 0.001), and poor outcomes or mortality (*p* < 0.001).

### 3.5. The Meta-Analysis of the Neurenteric Cyst According to the Extent of Resection 

Among the 138 patients with neurenteric cysts, 85 underwent total resection (65.9%), 14 had near-total excision (10.9%), and 30 had subtotal excision (23.3%). Age was not significantly different between patients, according to the extent of resection, and the mean age was 27.9 ± 15.75 years—total resection; 27.5 ± 11.94—near-total resection; and 36.5 ± 23.36 years—subtotal resection (*p* = 0.063). Total resection was significantly more common in patients who operated in the prone position, while subtotal resection was more frequent in those who operated in the sitting position (*p* = 0.036). Cysts with total resection were significantly less likely to adhere to surrounding brain tissue than those with near-total or subtotal resections (*p* < 0.001). While the frequency of postoperative meningitis was not significantly different according to the extent of resection (*p* = 0.353), patients with complications were significantly more associated with subtotal resection (*p* = 0.024).

### 3.6. The Meta-Analysis of Neurenteric Cyst According to Different Variables

Statistical analysis indicated that patients with CSF diversion were older (median age 43 years) compared to those without hydrocephalus or CSF diversion (median age = 27 years) (*p* = 0.010). The presence of rim enhancement on MRI (*p* = 0.030), use of the transoral approach (*p* < 0.001), postoperative meningitis (*p* = 0.039), complications (*p* < 0.001) and poor outcomes or mortality (*p* < 0.001) were significantly associated with CSF diversion.

The frequency of symptoms did not significantly differ according to age categories (*p* > 0.05), except for meningitis, which was more frequent in patients over 70 years (*p* = 0.026).

Statistical tests revealed that deceased patients had significantly higher ages compared to those with good (*p* = 0.012) or excellent outcomes (*p* = 0.015). Patients aged 0–4 years more frequently had good outcomes rather than excellent ones (*p* > 0.001), and patients with hydrocephalus more often had poor outcomes (*p* = 0.002). All of the results are available in the Supplementary Material (File S1).

## 4. Discussion

In reviewing the series of case reports, several notable similarities and differences emerge, shedding light on the diverse nature of intradural extramedullary tumors at the CVJ. All cases involved patients presenting with a spectrum of neurological symptoms, including headache, gait disturbance, and ataxia, indicative of the common clinical manifestations associated with such cysts. The consistent finding that surgical excision is the cornerstone of management underscores the critical role of timely and precise surgical intervention in addressing these complex lesions. However, the choice of surgical approach varied, reflecting the unique anatomical challenges and tumor characteristics in each case.

This systematic review is the first to comprehensively analyze clinical risk factors’ frequency and prognostic relevance in patients with tumor-like lesions at the CVJ. Given the limited number of cases involving dermoid or epidermoid cysts, our analytical findings predominantly focus on neurenteric cysts to ensure robust statistical validity. This review provides an updated synthesis of various clinical variables influencing the course of these pathologies, which is crucial for improving diagnostic accuracy and treatment strategies.

The most common tumors at the CVJ are intradural extramedullary tumors, such as meningiomas, neurofibromas, and schwannomas, while extradural tumors, including metastases and chordomas, are the second most frequent [3]. Over the past four decades, 170 tumor-like lesions have been reported at the CVJ, including the cases presented in this study. In our series of 48 CVJ cases treated over the last 12 years by a single surgeon (I. St. Florian), meningiomas were the most prevalent (20 cases), followed by schwannomas (4 cases), hemangioblastomas (2 cases), and ependymomas (2 cases).

To ensure accurate preoperative diagnosis and the appropriate management of cystic lesions at the craniovertebral junction (CVJ), it is essential to consider a broad range of differential diagnoses. These include intradural extramedullary tumors, such as meningiomas, schwannomas, and neurofibromas; intramedullary tumors, such as ependymomas, gliomas, and hemangioblastomas; and extradural tumors, including chordomas, chondrosarcomas, and osteosarcomas. Other conditions that may mimic these lesions include infectious cysts, such as neurocysticercosis and tuberculomas, as well as inflammatory cysts like sarcoidosis. Lipomas and arachnoid cysts are also considered in the differential diagnosis due to their unique imaging features. To aid in the differentiation of these conditions, we included a table in File S2 that summarizes the key radiological and histopathological characteristics of these lesions. This table is designed to provide clinicians with a comprehensive framework for evaluating and managing cystic lesions at the CVJ [1,2,3,4,16,17]. 

### 4.1. Tumor Types and Demographics

Among tumor-like lesions in the CVJ, neurenteric cysts constituted the most significant proportion at 81.2%; this is particularly surprising because, in intracranial locations, neurenteric cysts typically present the lowest proportion. The median age at presentation was 27 years for all patients. Still, dermoid cysts had a higher frequency in younger populations, with a median age of 16 years, consistent with their predilection for individuals under 18. The majority of symptoms manifested in younger patients, with a peak incidence in the 5–18 years age range (24.1%—40 cases), followed by 19–30 years (22.9%—38 cases), and 31–40 years (15.9%—27 cases). Only 15.3% of cases—26 cases—became symptomatic after age 50, indicating that these cysts typically present in young adulthood.

### 4.2. Clinical Implications

Our analysis highlights that those symptoms with a more significant clinical impact, such as headaches, neck pain, head and neck movement limitations, sensory abnormalities, and meningitis, are more commonly associated with dermoid cysts. This may be due to the frequent dissemination of cyst contents into the subarachnoid space, which occurs more often with dermoid cysts than neurenteric cysts. In contrast, gradually progressive neurological deficits such as paresis, cerebellar signs, lower cranial nerve paresis, and symptoms related to hydrocephalus are more frequently seen in patients with epidermoid cysts. This underscores the typically benign nature of epidermoid cysts, which often have a protracted symptom duration exceeding one year.

The literature often suggests that dermoid cysts have a midline predilection, whereas epidermoid cysts are more frequently paramedian. A notable observation from our analysis is that, according to the literature, the epidermoid cyst is the third most common pathology in the cerebello-pontine angle, whereas at the CVJ, the neurenteric cyst is the most common type of cyst-like tumor; the neurenteric cyst is less common than epidermoid or dermoid cysts in general intracerebral pathology [18,19]. Also, our study found that neurenteric cysts predominantly occupied the anterior portion of the CVJ, which is as per literature results [20,21,22,23]. In contrast, dermoid and epidermoid cysts were mainly located in the posterior CVJ, particularly at the cerebellomedullary cistern (*p* < 0.001). Cysts extending into the cervical spinal canal were significantly more associated with epidermoid cysts (*p* < 0.001). This characteristic illustrates the capacity of epidermoids to extend along the subarachnoid space, displacing, and compressing adjacent neural structures, such as the spinal cord, nerve roots, or blood vessels, without direct invasion or destruction. 

Furthermore, the study highlights the importance of accurate preoperative imaging and surgical planning in managing these lesions. Advanced MRI techniques, including DWI, provide crucial insights into these lesions’ cystic nature and extent, aiding in the differentiation from other pathologies. The selection of the surgical approach, whether far-lateral, transcondylar, retrosigmoid, or transoral, is guided by the cyst’s specific anatomical location and extension. Despite the high adhesion rate to surrounding brain tissue (70.7%—87 cases), complete excision was feasible in 63.7% of cases—100 cases, emphasizing the importance of meticulous surgical technique and intraoperative navigation.

### 4.3. Surgical Outcomes and Recurrence

The maximal surgical removal of the cystic lesion, dependent on the anatomic location and the condition of the patient, was recommended in all case reports. According to our study, gross total resection was feasible in 63.7% of cases—100 cases. Cyst recurrence was observed in 15 patients (12%), with 69.2% of recurrences—9 cases—occurring in cases of subtotal resection compared to 15.4% in cases—2 cases—of total resection (*p* < 0.001), with a median time to recurrence of 6 months. The presence of residual lesions was judged by postoperative MRI or CT scans; six cases with recurrence had a small residual lesion on imaging immediately postoperatively. One case developed extensive leptomeningeal dissemination three months after surgery [6,24,25,26,27,28]. Regarding recurrent lesions, re-surgery was applied in 10 cases, with 3 occurring within the first 6 months postoperatively and 7 occurring more than 3 years after the initial operation [6,18,27,29,30,31,32,33,34]. For the other five cases, no additional surgery was performed due to the poor general condition of the patient, advanced age, or a joint decision by the surgeon and patient to continue clinical-imaging follow-up for asymptomatic cases [6,25,26,28,35]. Although considered benign, malignant transformation is possible but very rare, reported in two cases [28,31] with the MT phenotype adenocarcinoma, one of which had papillary proliferation. The retrosigmoid region offers extensive access to the cerebellopontine angle, which is particularly beneficial for the excision of epidermoid cysts. The literature also supports the midline predilection of dermoid cysts at the CVJ, especially in its posterior portion, where a posterior midline approach is primarily utilized. This approach is also effective for managing neurenteric cysts located in the premedullary cistern and extending into the cervical spinal canal, with a total excision rate of 64.4%—38 cases. For lesions causing anterior compression of the cervicomedullary segment, a transoral approach was performed in four cases, with spinal fusion considered appropriate in half of these cases [13,29,36,37].

### 4.4. Complications and Management

Postoperative complications are neither few nor negligible and can occur despite meticulous surgical techniques and careful postoperative management. The most common complication was transient or permanent cranial nerve palsies (14 cases—9.27%), followed by postoperative hydrocephalus (11 cases—7.28%) requiring VP shunt or endoscopic third ventriculostomy. CSF fistula occurred in six patients—3.97%; for five, lumbar drainage was successful, with two also presenting cervical instability following a transoral approach necessitating a lumboperitoneal shunt [13,37,38]. The sixth case involved a CSF leak and Pseudomonas infection, treated with intravenous and intrareservoir antibiotic therapy, ultimately requiring a VP shunt [29]. Other complications included aseptic meningitis (nine cases), respiratory complications (three cases), pseudomeningocele (two cases), hematoma within a cavitary residual lesion (one case), and wound infection (one case).

According to the analysis, the transoral route should be approached with caution for tumor-like lesions due to its higher association with cervical instability, CSF leaks, and the need for CSF diversion compared to classic posterior approaches (*p* = 0.004, *p* = 0.001, *p* < 0.001, respectively). Associated anomalies frequently occur among patients with CVJ lesions, particularly congenital ones. In our study, 17.6% of cysts (29 cases) had associated anomalies, significantly more common in dermoid cysts (*p* < 0.001). These anomalies included the Klippel–Feil syndrome, dermal sinus tract, os odontoideum, bifid clivus, and spina bifida [1,18,30,39,40,41,42,43,44,45,46,47,48,49,50,51,52,53,54,55].

Patient outcomes following surgery at the CVJ depend on numerous factors, including a surgical technique, postoperative care, and patient-specific characteristics. Many reports have documented the resolution of neurological deficits, including quadriplegia, the postsurgical decompression of tumor-like lesions, with symptom-free intervals at six-month follow-ups in 75.2% of cases. Conversely, one patient had a poor prognosis, and seven patients died [25,28,31,32,33,34,56]. Therefore, the close monitoring and ongoing management are essential to optimize the outcomes and address challenges that may arise during recovery.

### 4.5. Meta-Analysis of the Neurenteric Cyst Located at the CVJ 

Analysis according to the existence of recurrence identified older age (*p* = 0.005) and the presence of postoperative complications (*p* < 0.001) as significant predictive factors for recurrence in neurenteric cysts. The extent of resection is a well-established prognosticator for recurrence [2,6,9,10,57,58,59,60,61,62,63,64,65], symptomatic hydrocephalus (*p* = 0.025) preceded recurrence due to mechanisms involving chemical meningitis, subsequent inflammation, and a granulomatous reaction in the arachnoid villi impairing CSF absorption. Clinically evident hydrocephalus necessitated CSF diversion (*p* = 0.001) [39,66,67].

Chronic inflammation, characterized by T1 hypointensity due to paramagnetic substances such as hemosiderin or inflammatory debris and rim enhancement on imaging studies, indicates areas of increased cellularity [27,61,68,69,70]. This process complicates the complete ablation of expansive lesions, as demonstrated in our study, where cysts with T1 hyposignal (*p* = 0.007) and rim enhancement (*p* = 0.002) were significantly associated with recurrence. Outcome frequencies differed significantly with recurrence (*p* < 0.001); patients with excellent outcomes were less likely to experience recurrence, while those with a poor prognoses or mortality had higher recurrence rates due to rapid disease progression, newly developed neurological deficits, or malignant degeneration. Our results in the multivariable analysis indicate that rim enhancement (*p* = 0.047) and poor outcome (*p* = 0.007) are significant factors associated with recurrence. Specifically, rim enhancement increases the odds of recurrence by 14.13 times (95% CI: 1.035–193.03), while patients with a poor outcome have their odds of recurrence increased by 139.07 times (95% CI: 3.76–5139.77), as detailed in Table 3.

Achieving complete resection while preserving neurological function is challenging, particularly for tumors located close to the brainstem, spinal cord, or major blood vessels. Surgeons often prioritize preserving neurological function over complete tumor removal, leading to subtotal resections in some cases. The interface between the cyst and normal neural tissue can be unclear, and the cyst may be very adherent to surrounding brain tissue, preventing the gross total removal [1,51,71,72]. In our study, cysts with total resection were significantly less associated with adhesion to surrounding brain tissue than those with near-total or subtotal resections (*p* < 0.001). The extensive manipulation of neural structures, dura mater, and surrounding tissues for total resection often leads to increased postoperative complications, which were more associated with subtotal resection (*p* = 0.024). 

According to the main surgeon’s experience, the sitting position significantly facilitates the total resection of CVJ lesions. This position allows gravity to aid in draining cyst contents and eases the dissection of vascular and nervous structures. As a result, most posterior fossa and CVJ pathologies are managed in this manner, a practice introduced in our clinic in 1975. However, surgery in the sitting position poses unique intraoperative challenges, including hemodynamic instability, venous air embolism, and surgical ergonomics. These challenges can impact the surgical precision, dexterity, and efficiency, potentially affecting the extent of resection and surgical outcomes. According to our meta-analysis, patients operated on in the prone position were significantly more associated with total resection (*p* = 0.036). Older adults may have age-related changes in immune function, decreased CSF turnover, or impaired CSF resorption mechanisms, predisposing them to CSF leaks and subsequent meningitis. Statistical analysis showed a significantly higher frequency of meningitis in patients over 70 years (*p* = 0.026).

### 4.6. Future Directions

Future research should focus on the long-term outcomes of different surgical approaches, the role of adjuvant therapies in preventing recurrence, and the genetic underpinnings of these tumor-like lesions. Long-term follow-up studies would provide more robust data on the natural history of these conditions and prospective clinical trials comparing different surgical approaches and techniques would help establish evidence-based guidelines for the management of CVJ cysts. Multi-center trials are needed to validate our findings and improve clinical guidelines. Collaborations across multiple institutions would enhance the generalizability of the results and help establish standardized treatment protocols.

## 5. Conclusions

The personal case series highlights the successful surgical management of CVJ cysts, including neurenteric, dermoid, and epidermoid cysts, using various surgical approaches. The findings emphasize the importance of individualized treatment strategies based on the cyst’s location and characteristics. Total resection was achieved in most cases, resulting in excellent long-term outcomes with minimal complications. The use of the sitting position for posterior fossa and CVJ pathologies facilitated the total resection by aiding in cyst drainage and improving access to vascular and nervous structures. However, leaving small fragments of the capsule in place was sometimes necessary due to the cyst’s adherence to critical structures. This underscores the need for meticulous surgical planning and technique to minimize recurrence and optimize patient outcomes.

In summary, the surgical management of tumor-like lesions at the CVJ is complex and requires a nuanced understanding of each case’s anatomical and pathological characteristics. Neurenteric cysts, dermoid cysts, and epidermoid cysts each present unique challenges that influence the surgical approach, extent of resection, and patient outcomes. This comprehensive review underscores the importance of individualized treatment plans, meticulous surgical techniques, and vigilant postoperative care to optimize outcomes and reduce recurrence rates.

Key findings from this meta-analysis highlight the critical role of gross total resection in reducing recurrence, the need for careful preoperative planning and imaging, and the potential complications associated with different surgical approaches. The presence of associated anomalies, the patient’s age and general health also significantly impact surgical outcomes and recurrence rates. Our study emphasizes the importance of long-term follow-up and the need for further research to refine surgical techniques and improve patient prognosis.

## Figures and Tables

**Table 2:** Characteristics of the analyzed cases.

Parameter (Nr., %)/Group	Total	Neurenteric	Dermoid	Epidermoid	*P*
Histopathology	170 (100%)	138 (81.2%)	19 (11.2%)	13 (7.6%)	-
Sex (male)	83 (48.8%)	66 (47.8%)	12 (63.2%)	5 (38.3%)	0.348 *
Age (median (IQR))	27 (15–42)	30 (16–41.25)	16 (7–31)	27 (16.5–57)	0.182 **
Signs and symptoms (Nr., %)					
Headache (N = 167)	95 (56.9%)	79 (58.5%)	7 (36.8%)	9 (69.2%)	0.141 *
Neck pain (N = 168)	59 (35.1%)	52 (38.2%)	5 (26.3%)	2 (15.4%)	0.198 *
Head and neck movement Limitation (N = 168)	29 (17.3%)	19 (14%)	7 (36.8%)	3 (23.1%)	0.032 *
Nausea and vomiting (N = 168)	26 (15.5%)	19 (14%)	4 (21.1%)	3 (23.1%)	0.461 *
*Paresis (N = 168)*					
Absent	119 (70.8%)	93 (68.4%)	16 (84.2%)	10 (76.9%)	0.590 *
Monoparesis	4 (2.4%)	4 (2.9%)	0 (0%)	0 (0%)	
Hemiparesis	9 (5.4%)	9 (6.6%)	0 (0%)	0 (0%)	
Quadriparesis	26 (15.5%)	22 (16.2%)	1 (5.3%)	3 (23.1%)	
Bibrachial paresis	4 (2.4%)	2 (1.5%)	2 (10.5%)	0 (0%)	
Paraparesis	2 (1.2%)	2 (1.5%)	0 (0%)	0 (0%)	
Other pyramidal signs	4 (2.4%)	4 (2.9%)	0 (0%)	0 (0%)	
6,7,8th nerve paresis (N = 168)	19 (11.3%)	14 (10.3%)	2 (10.5%)	3 (23.1%)	0.329 *
Lower cranial nerve paresis (N = 167)	29 (17.4%)	25 (18.5%)	3 (15.8%)	1 (7.7%)	0.741 *
Cerebellar signs (N = 167)	33 (19.8%)	19 (14.1%)	6 (31.6%)	8 (61.5%)	<0.001 *
Sensory abnormalities (N = 168)	29 (17.3%)	24 (17.6%)	5 (26.3%)	0 (0%)	0.135 *
Cardio-respiratory events (N = 167)	6 (3.6%)	6 (4.4%)	0 (0%)	0 (0%)	1.000 *
Bladder and bowel involvement (N = 168)	4 (2.4%)	3 (2.2%)	1 (5.3%)	0 (0%)	0.574 *
Meningitis (N = 168)	22 (13.1%)	15 (11%)	6 (31.6%)	1 (7.7%)	0.049 *
Symptomatic hydrocephalus (N = 168)	12 (7.1%)	7 (5.1%)	2 (10.5%)	3 (23.1%)	0.030 *
Nystagmus (N = 168)	9 (5.4%)	7 (5.1%)	1 (5.3%)	1 (7.7%)	0.811 *
Mirror movement (N = 168)	2 (1.2%)	0 (0%)	2 (10.5%)	0 (0%)	0.018 *
*Onset of symptoms (N = 144)*					
Insidious	126 (87.5%)	104 (86.7%)	12 (100%)	10 (83.3%)	0.468 *
Suddenly	18 (12.5%)	16 (83.3%)	0 (0%)	2 (16.7%)	
Symptoms duration (N = 125) (median (IQR)) (months)	5 (1.25–12)	4 (1–12)	6 (2–24)	10 (2.25–24)	0.315 **
*Location of tumor (N = 168)*					
Anterior CVJ	60 (35.7%)	56 (41.2%)	1 (5.3%)	3 (23.1%)	<0.001 *
Anterior and lateral CVJ	61 (36.3%)	57 (41.9%)	3 (15.8%)	1 (7.7%)	
Posterior CVJ	33 (19.6%)	12 (8.8%)	14 (73.7%)	7 (53.8%)	
Posterior and lateral CVJ	10 (6%)	7 (5.1%)	1 (5.3%)	2 (15.4%)	
Lateral CVJ	4 (2.4%)	4 (2.9%)	0 (0%)	0 (0%)	
*Cistern location (N = 167)*					
Premedullary cistern	25 (15%)	23 (17%)	0 (0%)	2 (15.4%)	<0.001 *
Premedullary cistern down to the spinal canal	43 (25.7%)	40 (29.6%)	3 (15.8%)	0 (0%)	
Cerebellomedullary cistern	23 (13.8%)	7 (5.2%)	13 (68.4%)	3 (23.1%)	
Cerebellomedullary cistern down to the spinal canal	18 (10.8%)	11 (8.1%)	2 (10.5%)	5 (38.5%)	
Pontomedullary cistern	21 (12.6%)	20 (14.8%)	0 (0%)	1 (7.7%)	
Pontomedullary cistern down to the spinal canal	18 (10.8%)	17 (12.6%)	0 (0%)	1 (7.7%)	
Subarachnoid space at the level of upper cervical canal	17 (10.2%)	16 (11.9%)	0 (0%)	1 (7.7%)	
Intramedullary	2 (1.2%)	1 (0.7%)	1 (5.3%)	0 (0%)	
Cyst volume (Median (IQR)) (N = 40)	14.86 (6.26–40.16)	13.87 (6.43–30)	8 (6.89–37.6)	54.9 (28.08–84.38)	0.254 **
*MRI aspect (N = 129)*					
Homogeneous	92 (71.3%)	74 (71.8%)	10 (66.7%)	8 (72.7%)	0.934 *
Heterogeneous	37 (28.7%)	29 (28.2%)	5 (33.3%)	3 (27.3%)	
*T1 aspect (N = 140)*					
Hyposignal	54 (38.6%)	39 (34.2%)	8 (57.1%)	7 (58.3%)	0.090 *
Isosignal	19 (13.6%)	18 (15.6%)	1 (7.1%)	0 (0%)	
Hypersignal	53 (37.9%)	46 (40.4%)	2 (14.3%)	5 (41.7%)	
Mixed	14 (10%)	11 (9.6%)	3 (21.4%)	0 (0%)	
*T2 aspect (N = 130)*					
Hyposignal	23 (17.7%)	16 (14.8%)	2 (20%)	5 (41.7%)	0.320 *
Isosignal	9 (6.9%)	8 (7.4%)	0 (0%)	1 (8.3%)	
Hypersignal	89 (69.5%)	76 (70.4%)	7 (70%)	6 (50%)	
Mixed	9 (6.9%)	8 (7.4%)	1 (10%)	0 (0%)	
*DWI (N = 36)*					
No restriction	30 (83.3%)	23 (88.5%)	1 (33.3%)	6 (85.7%)	0.020 *
Mild restriction	3 (8.3%)	3 (11.5%)	0 (0%)	0 (0%)	
Hyperintense	3 (8.3%)	0 (0%)	2 (66.7%)	1 (14.3%)	
*Enhancement (N = 108)*					
Absent	74 (68.5%)	61 (66.3%)	5 (83.3%)	8 (20%)	0.895 *
Rim enhancement	28 (25.9%)	25 (27.2%)	1 (16.7%)	2 (20%)	
Homogenous	4 (3.7%)	4 (4.3%)	0 (0%)	0 (0%)	
Other (irregular/linear)	2 (1.9%)	2 (2.2%)	0 (0%)	0 (0%)	
*Position (N = 60)*					
Lateral	23 (38.3%)	21 (40.4%)	1 (33.3%)	1 (20%)	0.058 *
Prone	21 (35%)	20 (38.5%)	1 (33.3%)	0 (0%)	
Supine	3 (5%)	3 (5.8%)	0 (0%)	0 (0%)	
Sitting	13 (21.7%)	8 (15.4%)	1 (33.3%)	4 (80%)	
*Craniotomy approach (N = 135)*					
Far-lateral	34 (25.2%)	31 (29%)	2 (11.1%)	1 (10%)	0.002 *
Far-lateral transcondylar	23 (17%)	22 (20.6%)	1 (5.6%)	0 (0%)	
Posterior midline	51 (37.8%)	30 (28%)	15 (83.3%)	6 (60%)	
Retrosigmoid	23 (17%)	20 (18.7%)	0 (0%)	3 (30%)	
Transoral	4 (3%)	4 (3.7%)	0 (0%)	0 (0%)	
Adhesion to surrounding brain (N = 123)	87 (70.7%)	72 (70.6%)	9 (69.2%)	6 (75%)	1.000 *
*Extent of resection (N = 157)*					
Total	100 (63.7%)	85 (65.9%)	9 (52.9%)	6 (54.5%)	0.452 *
Near-total	17 (10.8%)	14 (10.9%)	1 (5.9%)	2 (18.2%)	
Subtotal	40 (25.5%)	30 (23.3%)	7 (41.2%)	3 (27.3%)	
Postoperative meningitis	10 (5.9%)	10 (7.2%)	0 (0%)	0 (0%)	0.556 *
Complications (N = 151)	41 (27.2%)	35 (28%)	5 (31.3%)	1 (10%)	0.443 *
*Postoperative CT/MRI (N = 109)*					
No evidence of recurrence	96 (88.1%)	82 (86.3%)	8 (100%)	6 (100%)	0.653 *
Residual lesion	13 (11.9%)	13 (13.7%)	0 (0%)	0 (0%)	
Follow-up period (Median (IQR)) (N = 125)	22 (6–40.5)	14 (6–36)	32 (8.5–81)	24 (4.5–60)	0.313 **
Associated anomalies (N = 165)	29 (17.6%)	12 (9%)	15 (78.9%)	2 (16.7%)	<0.001 *
*Outcome (N = 149)*					
Excellent	112 (75.2%)	92 (75.4%)	12 (75%)	8 (72.7%)	0.836 *
Good	29 (19.5%)	22 (18%)	4 (25%)	3 (27.3%)	
Poor	1 (0.7%)	1 (0.8%)	0 (0%)	0 (0%)	
Dead	7 (4.7%)	7 (5.7%)	0 (0%)	0 (0%)	
Recurrence (N = 125)	15 (12%)	13(12.4%)	1 (8.3%)	1 (12.5%)	1.000 *
CSF diversion (N = 153)	24 (15.7%)	19 (14.6%)	3 (27.3%)	2 (16.7%)	0.462 *
Instrumentation (N = 161)	8 (5%)	5 (3.8%)	2 (11.8%)	1 (7.7%)	0.221 *

* Fisher’s exact test, ** Kruskal–Wallis H test.

**Table 3:** Logistic regression models used in predicting recurrence in patients with neurenteric cysts.

Parameter	Univariable		Multivariable *	
	OR (95% C.I.)	*p*	OR (95% C.I.)	*P*
Symptomatic hydrocephalus	8.80 (1.56–49.57)	0.014	0.49 (0.00–131,333.92)	0.490
T1-hyposignal	4.26 (1.13–15.96)	0.031	5.87 (0.35–97.83)	0.217
Rim enhancement	9.66 (2.11–44.15)	0.003	14.13 (1.035–193.03)	0.047
Subtotal resection	9.53 (2.62–34.65)	0.001	2.28 (0.15–32.93)	0.545
Poor outcome	43 (7.09–260.56)	<0.001	139.07 (3.76–5139.77)	0.007

* Omnibus test of model coefficient, X^2^ (5, N = 70) = 28.982 *p* < 0.001, Nagelkerke R^2^ = 0.666, Hosmer and Lemeshow test, *p* = 0.548, Sensitivity = 62.5%, Specificity = 96.8%, Overall accuracy = 92.9%.

## Data Availability

Not applicable.

## Supplementary Materials

The following supporting information can be downloaded at: https://www.mdpi.com/article/10.3390/cancers16162788/s1, File S1: The distribution of different variables in patients with neurenteric cysts. File S2: The differential diagnosis of CVJ tumor-like lesions: imaging and histopathological characteristics.
