# Supplementary material for: Tumor-Like Lesions in the Craniovertebral Junction: A Case Series, Systematic Review, and Meta-Analysis
Source: Cancers (Basel). 2024 Aug 7;16(16):2788. doi: 10.3390/cancers16162788 (PMC11352424; doi:10.3390/cancers16162788)
Supplement: Supplementary file 1 [file cancers-16-02788-s001.zip › S1 The distribution of different variables in patients with neurenteric cysts.pdf]

Distribution of Different Variables in Patients with Neurenteric Cysts

1. Recurrence

Parameter	Present	Absent	P-value
**Age category**			0.005*
**0-4 years**	2 (15.4%)	6 (6.5%)	
**5-18 years**	2 (15.4%)	22 (23.9%)	
**19-30 years**	1 (7.7%)	22 (23.9%)	
**31-40 years**	0 (0%)	17 (18.5%)	
**41-50 years**	2 (15.4%)	15 (16.3%)	
**51-60 years**	4 (30.8%)	8 (8.7%)	
**61-70 years**	0 (0%)	2 (2.2%)	
**71-80 years**	1 (7.7%)	0 (0%)	
**81-90 years**	1 (7.7%)	0 (0%)	
**Symptomatic hydrocephalus**	3 (23.1%)	3 (3.3%)	0.025*
**MRI T1 aspect**			0.007*
**Hyposignal**	7 (63.6%)	23 (29.1%)	
**Isosignal**	2 (18.2%)	12 (15.2%)	
**Hypersignal**	0 (0%)	36 (45.6%)	
**Mixed**	2 (18.2%)	8 (10.1%)	
**Enhancement**			0.002*
**Absent**	2 (22.2%)	53 (75.7%)	
**Rim enhancement**	6 (66.7%)	12 (17.1%)	
**Homogenous**	0 (0%)	4 (5.7%)	
**Other (irregular/linear)**	1 (11.1%)	1 (1.4%)	
**Extent of resection**			<0.001*
**Total**	2 (15.4%)	62 (69.7%)	
**Near-total**	2 (15.4%)	10 (11.2%)	
**Subtotal**	9 (69.2%)	17 (19.1%)	
**Complications**	12 (92.3%)	16 (18%)	<0.001*
**Outcome**			<0.001*
**Excellent**	2 (16.7%)	70 (79.5%)	
**Good**	4 (33.3%)	16 (18.2%)	
**Poor**	1 (8.3%)	0 (0%)	
**Dead**	5 (41.7%)	2 (2.3%)	
**CSF diversion**	8 (61.5%)	6 (6.5%)	<0.001*

*Fisher's Exact Test

2. Associated Anomalies

Parameter	Present	Absent	P-value
**Craniotomy approach**			0.049*
**Far-lateral**	0 (0%)	31 (31.6%)	
**Far-lateral transcondylar**	2 (28.6%)	19 (19.4%)	
**Posterior midline**	5 (71.4%)	24 (24.5%)	
**Retrosigmoid**	0 (0%)	20 (20.4%)	
**Transoral**	0 (0%)	4 (4.1%)	

*Fisher's Exact Test

3. **CSF Diversion**

Parameter	Absent	Present	P-value
**Age (Median (IQR))**	27 (16-40)	43 (31-57)	0.010**
**Age category**			0.001*
**0-4 years**	7 (6.3%)	3 (15.8%)	
**5-18 years**	29 (26.1%)	0 (0%)	
**19-30 years**	28 (25.2%)	1 (5.3%)	
**31-40 years**	21 (18.9%)	4 (21.1%)	
**41-50 years**	13 (11.7%)	5 (26.3%)	
**51-60 years**	10 (9%)	5 (26.3%)	
**61-70 years**	2 (1.8%)	0 (0%)	
**71-80 years**	0 (0%)	1 (5.3%)	
**81-90 years**	1 (0.9%)	0 (0%)	
**Meningitis**	8 (7.3%)	6 (31.6%)	0.007*
**Symptomatic hydrocephalus**	2 (1.8%)	5 (26.3%)	0.001*
**Enhancement**			0.030*
**Absent**	56 (72.7%)	4 (36.4%)	
**Rim enhancement**	15 (19.5%)	7 (63.6%)	
**Homogenous**	4 (5.2%)	0 (0%)	
**Other (irregular/linear)**	2 (2.6%)	0 (0%)	
**Position**			0.001*
**Lateral**	17 (38.6%)	4 (50%)	
**Prone**	20 (45.5%)	0 (0%)	
**Supine**	0 (0%)	3 (37.5%)	
**Sitting**	7 (15.9%)	1 (12.5%)	
**Craniotomy approach**			<0.001*
**Far-lateral**	29 (31.2%)	2 (15.4%)	
**Far-lateral transcondylar**	19 (20.4%)	2 (15.4%)	
**Posterior midline**	28 (30.1%)	2 (15.4%)	
**Retrosigmoid**	17 (18.3%)	3 (23.1%)	
**Transoral**	0 (0%)	4 (30.8%)	
**Postoperative meningitis**	6 (5.4%)	4 (21.1%)	0.039*
**Complications**	19 (18.3%)	16 (84.2%)	<0.001*
**Outcome**			<0.001*
**Excellent**	84 (83.2%)	5 (29.4%)	
**Good**	14 (13.9%)	7 (41.2%)	
**Poor**	0 (0%)	1 (5.9%)	
**Dead**	3 (3%)	4 (23.5%)	

*Fisher's Exact Test, **Mann-Whitney U Test

4. ***Instrumentation***

Parameter	Present	Absent	P-value
**Craniotomy approach**			0.004*
**Far-lateral**	0 (0%)	31 (30.4%)	
**Far-lateral transcondylar**	1 (25%)	20 (19.6%)	
**Posterior midline**	1 (25%)	29 (28.4%)	
**Retrosigmoid**	0 (0%)	20 (19.6%)	

**Transoral**	2 (50%)	2 (2%)	

*Fisher's Exact Test

5. *CSF Leakage*

Parameter	Absent	Present	P-value
**Craniotomy approach**			<0.001*
**Far-lateral**	30 (29.4%)	1 (20%)	
**Far-lateral transcondylar**	22 (21.6%)	0 (0%)	
**Posterior midline**	30 (29.4%)	0 (0%)	
**Retrosigmoid**	19 (18.6%)	1 (20%)	
**Transoral**	1 (1%)	3 (60%)	

*Fisher's Exact Test

6. *Grade of Resection*

Parameter	Total Resection	Near-total Resection	Subtotal Resection	P-value
**MRI T1 aspect**				0.020*
**Hyposignal**	27 (37%)	0 (0%)	12 (50%)	
**Isosignal**	15 (20.5%)	2 (18.2%)	1 (4.2%)	
**Hypersignal**	26 (35.6%)	7 (63.6%)	8 (33.3%)	
**Mixed**	5 (6.8%)	2 (18.2%)	3 (12.5%)	
**Position**				0.036*
**Lateral**	13 (37.1%)	3 (42.9%)	5 (50%)	
**Prone**	17 (48.6%)	3 (42.9%)	0 (0%)	
**Supine**	2 (5.7%)	0 (0%)	1 (10%)	
**Sitting**	3 (8.6%)	1 (14.3%)	4 (40%)	
**Adhesion to surrounding brain**	35 (53.8%)	11 (100%)	26 (100%)	<0.001*
**Complications**	16 (20.3%)	6 (42.9%)	12 (44.4%)	0.024*

*Fisher's Exact Test

7. *Outcome*

Parameter	Excellent	Good	Poor	Dead	P-value
**Age (Median (IQR))**	26.5 (15-39.7)	20 (1.75-50)	78	44 (41-57)	0.005**
**Age category**					<0.001*
**0-4 years**	5 (5.4%)	6 (27.3%)	0 (0%)	0 (0%)	
**5-18 years**	27 (29.3%)	4 (18.2%)	0 (0%)	0 (0%)	
**19-30 years**	22 (23.9%)	5 (22.7%)	0 (0%)	0 (0%)	
**31-40 years**	18 (19.6%)	0 (0%)	0 (0%)	1 (14.3%)	
**41-50 years**	13 (14.1%)	2 (9.1%)	0 (0%)	3 (42.9%)	
**51-60 years**	7 (7.6%)	3 (13.6%)	0 (0%)	3 (42.9%)	
**61-70 years**	0 (0%)	1 (4.5%)	0 (0%)	0 (0%)	
**71-80 years**	0 (0%)	0 (0%)	1 (100%)	0 (0%)	
**81-90 years**	0 (0%)	1 (4.5%)	0 (0%)	0 (0%)	
**Symptomatic hydrocephalus**	2 (2.2%)	3 (14.3%)	1 (100%)	1 (14.3%)	0.002*

*Fisher's Exact Test, **Kruskal-Wallis H Test

- Age was significantly different between outcome groups (p=0.005) and post-hoc Dunn-Bonferroni tests show that dead patients (median = 44, IQR = 41-57) had a significantly higher age than patients with good outcome (median = 20, IQR = 1.75-50) (p=0.012) or excellent outcome (median = 26.5, IQR = 15-39.75) (p=0.015);

8. *Signs and Symptoms*

**Parameter**	**0-4 years**	**5-18 years**	**19-30 years**	**31-40 years**	**41-50 years**	**51-60 years**	**61-70 years**	**71-80 years**	**81-90 years**	**P-value**
**Meningitis**	4 (40%)	2 (6.7%)	2 (6.3%)	4 (16%)	2 (10.5%)	0 (0%)	0 (0%)	1 (100%)	0 (0%)	0.026*

*Fisher's Exact Test
