# Supplementary material for: Tumor-Like Lesions in the Craniovertebral Junction: A Case Series, Systematic Review, and Meta-Analysis
Source: Cancers (Basel). 2024 Aug 7;16(16):2788. doi: 10.3390/cancers16162788 (PMC11352424; doi:10.3390/cancers16162788)
Supplement: Supplementary file 1 [file cancers-16-02788-s001.zip › S2 The differential diagnosis of CVJ tumor-like lesions imaging and histopathological characteris-tics.pdf]

Differential Diagnosis of CVJ Tumor-like Lesions: Imaging and Histopathological Characteristics

Conditions of the CVJ	Radiological MRI Features	Histopathological Features
Intramedullary tumors		
Ependymoma	- Well-circumscribed, enhancing mass within the spinal cord;<br>- Often associated with cysts or syrinx;<br>- Heterogeneous signal intensity.	- Perivascular pseudorosettes and true rosettes;<br>- Ependymal cell differentiation.
Glioma	- Infiltrative, ill-defined mass within the spinal cord;<br>- Variable signal intensity on MRI;<br>- Often with contrast enhancement and surrounding edema.	- Astrocytic,oligodendroglial, or mixed glial cell types;<br>- Variable degrees of differentiation and mitotic activity.
Hemangioblastoma	- Well-circumscribed, cystic mass with an isointense nodule with prominent "flow voids" is seen on T1WI and moderately hyperintense on T2WI and FLAIR images;<br>- If an associated peritumoral cyst is present, it is typically hypointense to parenchyma on T1WI but hyperintense compared with CSF;<br>- Intense enhancement of the nodule.	- Capillary-rich vascular tumor;<br>- Presence of stromal cells;<br>- Lack of Necrosis.
Intradural extramedullary tumors		
Meningioma	- Well-circumscribed, iso- to slightly hypointense compared with cortex on T1WI and iso- to moderately hyperintense compared with cortex on T2WI;<br>- Dural tail sign often present (60%);<br>- Strong, often heterogeneous enhancement (> 98%).	- Depending on the histological subtype;<br>- „Mushroom cap" - morphology most common (75%).
Schwannoma	- Isointense with cortex on T1WI; heterogeneously hyperintense on T2WI & FLAIR;<br>- Areas of cystic degeneration;<br>- Contrast enhancement.	- Spindle cells arranged in Antoni A (cellular) and Antoni B (loose) patterns;<br>- Palisaded arrangement of nuclei in Antoni A regions.
Neurofibroma	- Well-circumscribed mass;<br>- Isointense or hypointense on T1WI, hyperintense on T2WI;<br>- Minimal to moderate enhancement.	- Composed of spindle cells with wavy nuclei;<br>- Presence of collagen bundles;<br>- Mixed cellularity.
Extradural tumors		
Chordoma	- Lobulated, destructive lesions;<br>- Heterogeneous signal intensity;<br>- Enhancement present.	- Physaliphorous cells within a myxoid stroma;<br>- Chondroid Areas.
Chondrosarcoma	- Lobulated mass with chondroid matrix calcification;<br>- Heterogeneous signal intensity;<br>- Moderate to intense enhancement.	- Malignant chondrocytes within a cartilaginous matrix;<br>- Significant atypia and pleomorphism of chondrocytes.
Osteosarcoma	- Heterogeneous mass with poorly defined margins, typically low to intermediate signal intensity on T1WI and high signal intensity in the areas of the tumor due to the presence of osteoid matrix and necrotic tissue on T2WI;<br>- Heterogeneous enhancement.	- Malignant osteoid production;<br>- Presence of osteoblastic cells.<br>- Spindle-shaped cells with marked atypia.
Metastases	- Variable signal intensity on T1 and T2;<br>- Multiple, well-defined enhancing lesions with surrounding edema;<br>- Can be also intra or extra-axial.	- Malignant cells consistent with the primary malignancy.
Other conditions		
Infectious Cysts	- Neurocysticercosis: Cyst with hyperintense rim on T1WI, hypointense rim with hyperintense center on T2WI and peripheral enhancement with contrast<br>- Tuberculoma: Hypointense on T1WI, isointense or hyperintense on T2WI, ring-enhancing lesions with contrast	- Neurocysticercosis: Larval form of Taenia solium, scolex may be visible<br>- Tuberculoma: Caseating granulomas with central necrosis.
Inflammatory Cysts	- Sarcoidosis: Enhancing masses or nodules with perilesional edema;	- Non-caseating granulomas with multinucleated giant cells (Sarcoidosis).
Lipoma	- High signal intensity on T1WI and low signal intensity on T2WI;<br>- No enhancement with contrast;<br>- Suppressed signal on fat-saturation sequences.	- Composed of mature adipocytes;<br>- Adipocytes arranged in lobules separated by fibrous septa.
Arachnoid Cyst	- Follows cerebrospinal fluid (CSF) signal intensity on all MRI sequences.<br>- No enhancement.	- Lined by arachnoid cells;<br>- Thin, Delicate Wall: Composed of collagen and arachnoid cell layers.
